# Human hair keratins promote the regeneration of peripheral nerves in a rat sciatic nerve crush model

**DOI:** 10.1007/s10856-019-6283-1

**Published:** 2019-07-04

**Authors:** Jianyi Gao, Lei Zhang, Yusheng Wei, Tianyan Chen, Xianyan Ji, Kai Ye, Jiahong Yu, Bin Tang, Xiaochun Sun, Jiabo Hu

**Affiliations:** Jinagsu Key Laboratory of Medical Science and Laboratory Medicine, School of Medicine, Jiangsu University, 301 Xuefu Road, Zhenjiang, 212013 Jiangsu China

## Abstract

Axon regeneration and functional recovery after peripheral nerve injury remains a clinical challenge. Injury leads to axonal disintegration after which Schwann cells (SCs) and macrophages re-engage in the process of regeneration. At present, biomaterials are regarded as the most promising way to repair peripheral nerve damage. As a natural material, keratin has a wide range of sources and has good biocompatibility and biodegradability. Here, a keratin was extracted from human hair by reducing method and a keratin sponge with porous structure was obtained by further processing. The results suggested that keratin can promote cell adhesion, proliferation, migration as well as the secretion of neurotrophic factors by SCs and the regulation of the expression of macrophage inflammatory cytokines in vitro. We report for the first time that human hair keratin can promote the extension of axon in DRG neurons. The motor deficits caused by a sciatic nerve crush injury were alleviated by keratin sponge dressing in vivo. Thus, keratin has been identified as a valuable biomaterial that can enhance peripheral nerve regeneration.

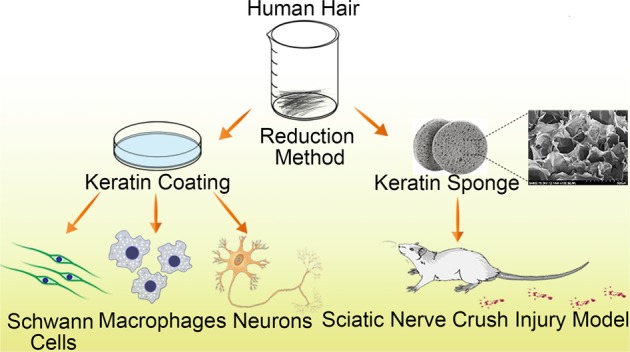

## Introduction

Peripheral nerve injury has been rising every year and caused a heavy economic burden on patients and society [1, 2]. Repair after injury is a complex and long process during which denervated Schwann cells (SCs) dedifferentiate into a more active phenotype, migrate to the damage site to form a Bungner belt, and then secrete neurotrophic factors to nourish axons, leading them to extend along the Bungner belt [3–5]. Macrophages participate in postinjury repair by clearing myelin fragments and expressing various inflammatory factors that regulate local inflammatory responses [6, 7].

To date, there is no effective treatment for peripheral nerve injury, and biomaterials are considered a promising option. In recent years, a variety of nerve repair materials have been developed, such as medical regenerative collagen membranes, polyglycolic acid and polylactic acid copolymers [8]. However, the application of these biological materials in the body still has diverse problems, including immune rejection, uncertainty of degradation speed, toxicity of degradation products, limited sources of materials and high costs [9, 10].

Human hair keratin is considered to be an excellent biomaterial to promote the repair of peripheral nerve injury due to its good biocompatibility, nonimmune response, good intercellular interaction and biodegradability [11, 12]. The adhesion sequences LDV and RGD of keratin are also found in several extracellular matrix proteins capable of supporting cell attachment [13–15]. Previous studies have shown that 12 kinds of cells, such as neural and bone marrow stem cells, have obvious proliferation and growth in cell culture membranes prepared with keratin nanoparticles [14]. Keratin can also form a uniform pore size keratin sponge under a certain condition and will maintain its shape for several hours at a high temperature of 60°C. This property distinguishes the keratin from relatively low denaturation temperature collagen [10, 13].

In this study, we hypothesized that keratin can activate repair-related cells in vitro and that a prepared keratin sponge, as a dressing, can accelerate the repair of sciatic nerve injury.

## Experimental materials and methods

### Human hair keratin extraction and fabrication of keratin sponge

The extraction of human hair keratin was performed by a previously reported method, with modifications [13]. Briefly, human hair keratin was extracted by reducing method. First, a mixture of chloroform and methanol (2:1 v/v) was added to the cleaned hairs for 15–20 min to degrease. Then the delipidized hairs (10 g) were added to a mixed solution prepared by the dissolution of SDS (6 g), urea (90 g) and β-mercaptoethanol (15 mL) in 300 mL double distilled water, to react at 60 ℃ for 3 days under the condition of pH 7. The resulting mixture was centrifuged and filtered through filter paper. The filtrate was then thoroughly dialyzed with double distilled water that would be replaced every 6 h until it completely removed urea, etc. The keratin filtered by 0.22 µm filter can be stored stably at room temperature for one year.

A total of 1 mL keratin (40 mg/mL) was added to each well of a 24-well plate, frozen at −20 °C for 3 days and then lyophilized to form a keratin sponge, which is stable and insoluble in water. Before it was used, the keratin sponge was sterilized with 70% ethanol for 2 h and equilibrated with Dulbecco’s Modified Eagle’s Medium (DMEM; Gibco, USA), supplemented with 10% fetal bovine serum (FBS; Gibco, USA).

### Electron microscopic (EM) analysis

The keratin sponge was cut into 1–2 mm thin slices and coated with Au prior to scanning electron microscopy (SEM; S-4800II FESEM, Japan).

The nerve samples were first fixed in 2.5% glutaraldehyde solution, rinsed in phosphate buffered saline (PBS; pH 7.3), fixed with 1% osmium tetroxide and dehydrated in a series of graded ethanol. The samples were embedded in Epon propylene oxide and incubated at 60 °C for 48 h. The ultrathin sections prepared and stained with lead citrate were observed under a transmission electron microscopy (TEM; HITACHI-HT7700, Japan). The average myelinated axon diameter and myelin sheath thickness were measured using ImageJ software (NIH, Bethesda, MD).

### Keratin coating

The sterile keratin (0.3 mg/mL) was added to the culture plate to cover the bottom and then placed in a 37 °C incubator for coating. After 24 h, the solution was aspirated to leave a thin fluid film on the bottom surface of wells.

### Cells culture

All the Sprague-Dawley (SD) rats were obtained from the Animal Experimental Center of Jiangsu University, and all experimental procedures were conducted with the approval of the Ethics Committee of Jiangsu University in accordance with Chinese legislation on animal protection.

The RSC96 (rat Schwann cell strain) and RAW264.7 cell lines were purchased under a material transfer agreement from the Stem Cell Bank, Chinese Academy of Sciences. The cell lines were cultured in DMEM supplemented with 10% FBS at 37 °C in a humidified atmosphere containing 5% CO_2_. The culture medium was changed every 2 days.

### Primary cells culture

Primary SCs were isolated from sciatic nerves of postnatal 1–3 days SD rats [16]. Cells were cultured on poly-L-lysine-coated (PLL; Yuanye Biotech, China) culture plates for 24 h and further purified by cytarabine (10 μM, Macklin Biochemical Co, China) for 48 h. P3-P8 primary SCs were used in this experiment.

Briefly, dorsal root ganglia (DRG) were obtained from three-day-old SD rats [17]. The isolated ganglia were incubated in 3 mg/mL collagenase A (Sigma, USA) for 30 min at 37 °C, and then 0.25% trypsin (Thermo Fisher, USA) was added for 30 min after neutralization and centrifugation. Finally, the single DRG was seeded in two groups for culture for 24 h.

### Cell adhesion assay

Keratin was coated on the right side of a 75 mm low adhesion polyethylene plate (Xinkang Medical, China). Then 5 × 10^7^ RSC96 cells were added and placed in a 37 ℃ incubator, and the adhesion of cells were observed on both sides after 24 h.

In order to further test the adhesion rate, 6 × 10^5^ RSC96 cells were added to the coated and uncoated standard polystyrene 24-well plate. Within 6 h of seeding, the number of unattached cells was counted per hour in both groups.

### Cell proliferation assay

Cells were seeded on coated and uncoated 96-well plates (3000 cells/well) supplemented with 1% FBS. Cell proliferation on days 1, 3, 5, and 7 was assessed by a Cell Counting Kit-8 (MCE, China). The absorbance was measured at 450 nm using a Microplate Reader (Bio-Rad, Hercules, USA).

Overall 5 × 10^6^ RSC96 cells were seeded on coated and uncoated 6-well plates supplemented with 10% FBS for 1, 3, 5, and 7 days. Then cells were stained with the hematoxylin and eosin (HE) kit (Shanghai Jinsui Bio-Technology Co., Ltd, China) according to the manufacturer’s instructions. Images were collected with an upright microscope.

### Cell migration assay

Migration of SCs was studied using 6.5 mm Transwell chambers with 8 µm pores (Costar, USA) as previously described [18]. DMEM (600 µL) with 10% FBS was added to the coated and uncoated lower chambers. Serum-free cell resuspension solution (200 µL) containing 5 × 10^4^ primary SCs was added to the upper chambers and cultured for 12 h. The chambers were removed and fixed with 4% paraformaldehyde for 30 min and stained with crystal violet after washing three times with PBS. The stained cells were imaged and counted under an upright microscope.

### Wound-healing assay

For the wound-healing assay, 5 × 10^4^ primary SCs were seeded on coated and uncoated 24-well plates. Cells were grown to a monolayer and serum-starved for 12 h. Then, a sterile 10 µL pipette tip was used to form a linear scratch and the medium of the 24-well plates was changed to serum-free medium immediately. The scratch areas were photographed with an inverted microscope at 0 and 12 h after wounding.

### mRNA expression analysis

The RSC96 and RAW264.7 cells were seeded on coated and uncoated 6-well plates. Total cellular RNA was extracted by Trizol reagent (Sigma, USA). According to the manufacturer’s instructions, the cDNA was obtained by One-Step gDNA Removal and cDNA Synthesis kit (Transgen Biotech, China), diluted 5-fold for real-time qPCR and analyzed with StepOnePlus™ (ABI). The relative expression intensity was obtained by calculating the 2^−△△Ct^ of each sample. The genes and the related specific primers used are shown in Table 1.

**Table 1:** Genes and oligonucleotide primers used in PCR analysis

Gene	Forward primer	Reverse primer
IL-1β	TGTGTTTTCCTCCTTGCCTCTGAT	TGCTGCCTAATGTCCCCTTGAAT
IL-6	AGGAGTGGCTAAGGACCAAGACC	CTGACCACAGTGAGGAATGTCCAC
i-NOS	AGCCTCTTGTCTTTGACCC	GAATCTTGGAGCGAGTTGTG
TNF-α	GCGACGTGGAACTGGCAGAAG	GCCACAAGCAGGAATGAGAAGAGG
IL-10	GGAAGACAATAACTGCACCCACT	AACCCAAGTAACCCTTAAAGTCC
β-actin	GTGCTATGTTGCTCTAGACTTCG	ATGCCACAGGATTCCATACC
BDNF	GTGGGTCACAGCGGCAGATA	ATGGGATTACACTTGGTCTCGTAG
CNTF	TGAGATGACTGAGGCAGAGCG	CTGGTAGGCAAAGGCAGAAAC
GDNF	AAATGTCACTGACTTGGGTTTGG	ACCTTGTCACTTGTTAGCCTTCTACT
NGF	AGCGTAATGTCCATGTTGTTCTACA	GTTTAGTCCAGTGGGCTTCAGG
VEGF	TGTGGACTTGAGTTGGGAGGAG	TGGCAGGCAAACAGACTTCG
β-actin	ACAACCTTCTTGCAGCTCCTC	CTGACCCATACCCACCATCAC

### Immunofluorescence (IF) staining

The DRG neurons were fixed with 4% paraformaldehyde for 20 min and washed three times with PBS for 5 min. Blocking solution with 2% bovine serum albumin (BSA) and 0.1% Triton-X100 by PBS was added to each well at 37 °C for 2 h to penetrate and incubate. DRG neurons were incubated with the primary antibody, rabbit anti-βIII-tubulin (1:300, Applied Biological Materials Inc., Canada), overnight at 4 °C. The next day, DRG neurons were incubated with Cy^TM3^-conjugated AffiniPure goat anti-rabbit IgG(H + L) (1:600, Jackson) for 2 h at 37 °C. Finally, DRG neurons were observed under an Olympus inverted fluorescence microscope (IX73, Olympus Corporation, USA).

### Rat sciatic nerve crush injury model

Male SD rats weighing 180–200 g were placed in a sterile environment. Twelve SD rats were randomly divided into two groups (n = 6 per group): (1) control group and (2) keratin group. The animals were completely anesthetized with pentobarbital (50 mg/kg) intraperitoneally, and the gluteal muscles were separated by blunt muscle separation to expose the right sciatic nerve. The middle segment of nerve was tightly crushed by hemostatic forceps for 30 s until its color became translucent. The prepared keratin sponge was wrapped around the damaged nerve, and the wound was closed in layers.

### Sciatic functional index

The sciatic functional index (SFI) was calculated and footprint analysis of SD rats was performed at 3, 7, 14, 17, and 21 days after surgery to assess the recovery of hind limb function. Nontoxic finger paint was applied to their hind limbs before they walked on the track to collect at least five measurable footprints. The normal side (N) and the experimental side (E) were measured separately, and the related parameters were as follows: the entire plantar length (PL), the distance from the first to fifth toes, the toe spread (TS), the distance between the second and fourth toes and the intermediary toe spread (IT). The SFI was calculated according to the following formula:

$${\mathrm{SFI = }} - {\mathrm{38}}{\mathrm{.3}} \times \left( {{\mathrm{EPL - NPL}}} \right){\mathrm{/NPL + 109}}{\mathrm{.5}} \times \left( {{\mathrm{ETS - NTS}}} \right){\mathrm{/NTS + 13}}{\mathrm{.3}} \times \left( {{\mathrm{EIT - NIT}}} \right){\mathrm{/NIT - 8}}{\mathrm{.8}}$$

SFI reflects the recovery of peripheral nerves. A score of −100 represents the total loss of function whereas 0 indicates an efficient nerve.

### Statistical analysis

All values are shown as the means ± SEM. Unpaired student’s *t*-tests were used to compare the differences between two groups. One-way ANOVA was used to compare the differences among all the groups and LSD-*t* test was used for further comparison between the two groups. Each experiment was independently performed at least three times. *P*-value < 0.05 was statistically significant.

## Results

### Biological characteristics of keratin

**Fig. 1:**
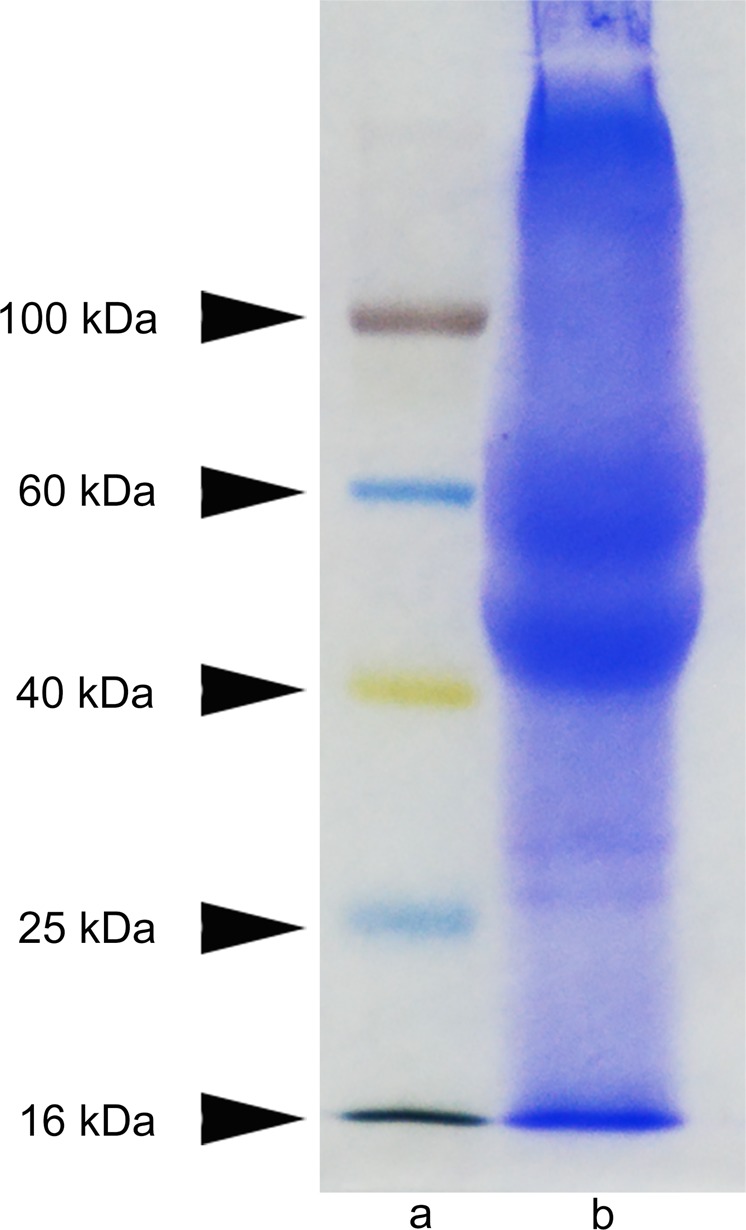
SDS-PAGE of the extracted human hair keratin. **a** Molecular weight marker. **b** Human hair keratin extract

Most of the keratin extracted by reduction method is α-keratin, which is mainly composed of microfibril keratins with a molecular weight of between 40 and60 kDa (Fig. 1b).

**Fig. 2:**
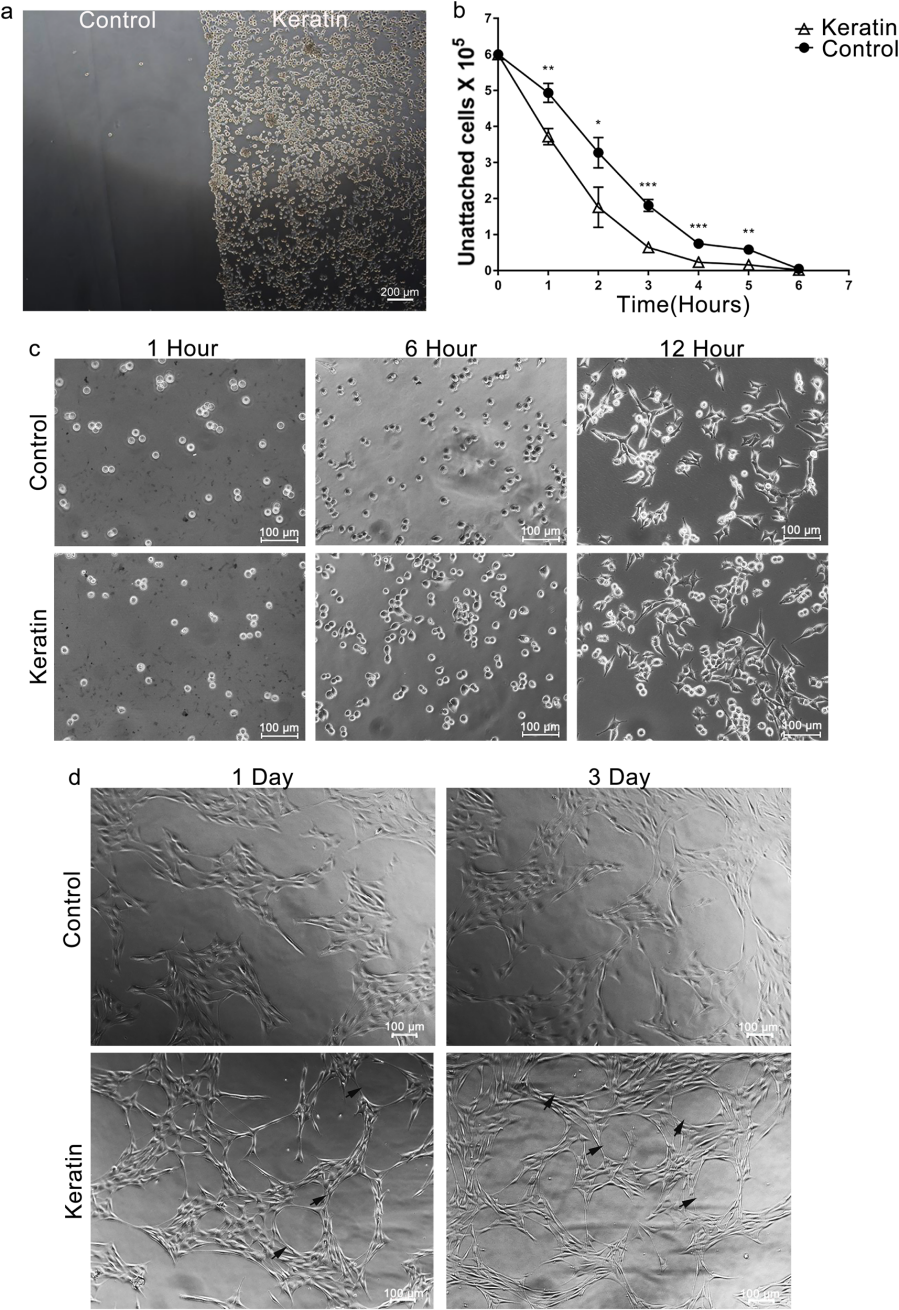
Adhesion and morphological assays of SCs. **a** Adhesion of RSC96 cells on keratin-coated and uncoated low adhesion polyethylene plates. **b** The number of unattached RSC96 cells after seeding were measured by count analysis within 6 h in two groups. **c** The morphologies of RSC96 cells in two groups were observed at 1, 6, and 12 h. **d** The morphologies of primary SCs in two groups were observed at days 1 and 3. The cell rings in the keratin group are shown by black arrowheads. *P *<* 0.05, **P < 0.01, ***P < 0.001 vs. the control group (**a** scale bars = 200 μm; **c**, **d** scale bars = 100 μm)

RSC96 cells only adhered to the right side of pre-coated low adhesion plate (Fig. 2a), suggesting that keratin has a good cell compatibility.

To evaluate the seeding efficiency, RSC96 cells were seeded on two groups at the same time. In the first 2 h, cells adhered quickly (~70.8% of cells) in the keratin group while the control group adhered only ~44.8% of cells (*P* < 0.05). With the prolongation of adherent time, the difference gradually decreased after 4 h, and the cells in two groups were completely adherent at 6 h (Fig. 2b). This showed that keratin is more helpful to cells adhesion.

Except for the change in adhesion rate, the morphology of RSC96 cells in keratin group began to change 12 h after seeding. The number of round cells were reduced and most of them turned into typical fusiform, spindle-like morphology (Fig. 2c). This phenomenon was more obvious in primary SCs, and the primary SCs of keratin group presented longer neurofilaments and formed rings in contact with each other after having been planted for 24 h. The number of cell-rings increased at 72 h more in the keratin group than in the control group (Fig. 2d).

### Effects of keratin on SCs and DRG

**Fig. 3:**
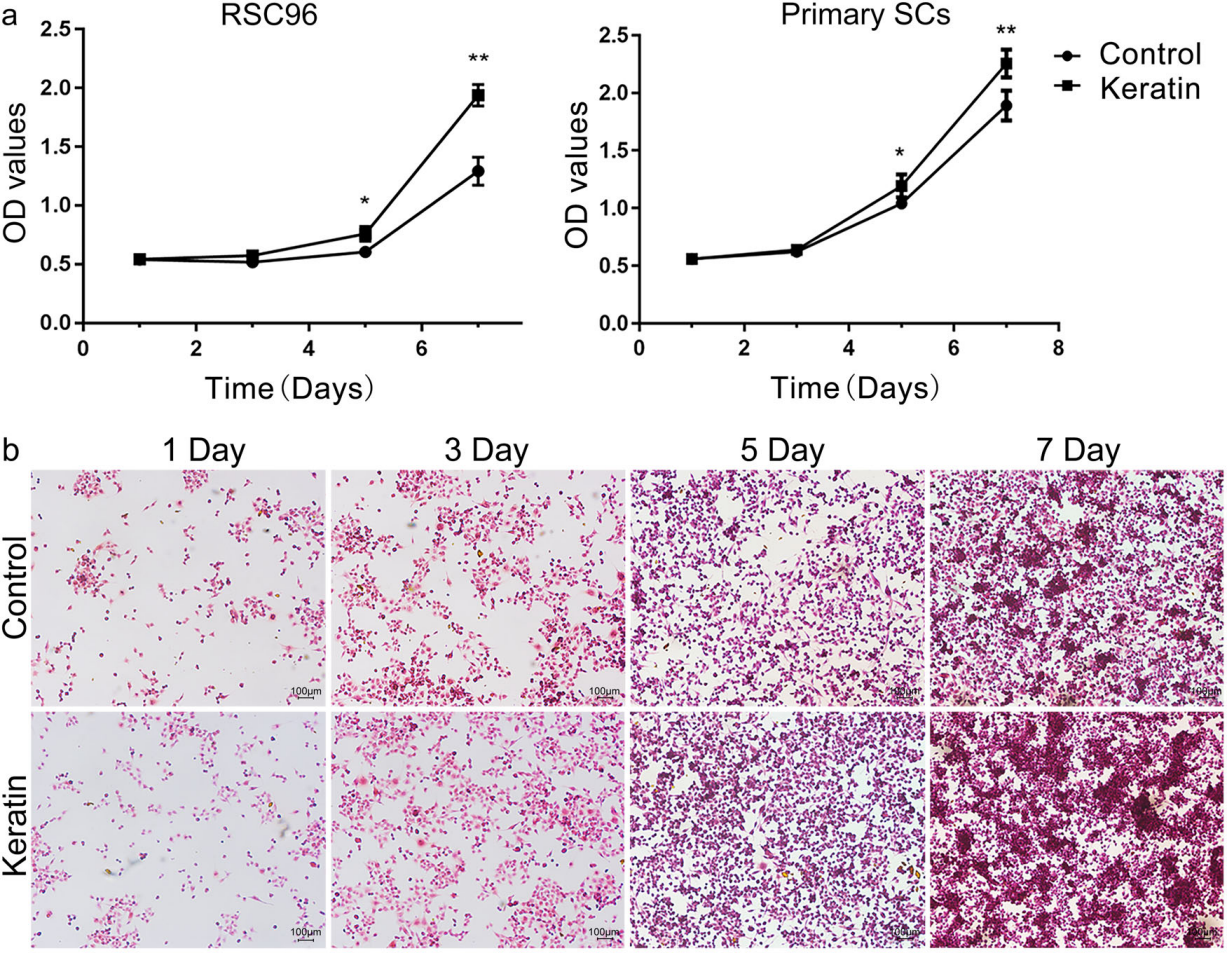
Proliferation of SCs in the keratin group and control group. **a** Proliferation of SCs in the keratin group and control group was evaluated by CCK-8 assay at days 1, 3, 5, and 7. **b** HE staining of RSC96 cells cultured in two groups for days 1, 3, 5 and 7. *P < 0.05, **P < 0.01 (**b** scale bars = 100 μm)

Compared with the control group, proliferation of RSC96 cells obviously increased on days 5 (*P* < 0.05) and 7 (*P* < 0.01) in keratin group. Due to the different affinity of keratin for various cells, primary SCs in keratin group began to proliferate after 3 days (Fig. 3a). More intuitive observation by HE staining showed that the intercellular space of RSC96 cells in keratin group was reduced on day 5, and more colony formations with cells piled onto one another by day 7. But this phenomenon was not obvious in control group (Fig. 3b).

**Fig. 4:**
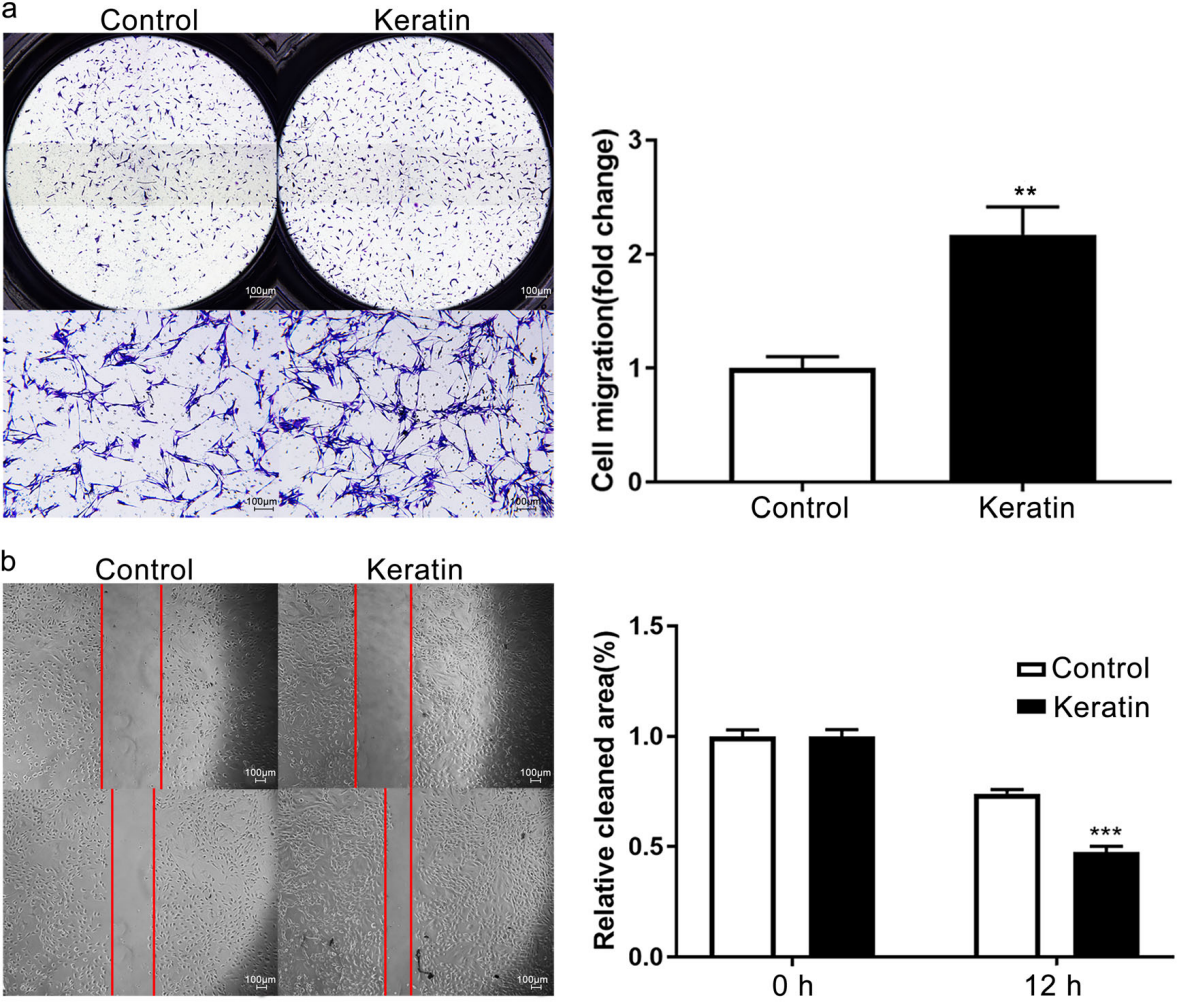
Keratin is associated with the migration of primary SCs. **a** Transwell assays of primary SCs in control and keratin groups. **b** Wound-healing assays of primary SCs in control and keratin groups. **P < 0.01, ***P < 0.001 vs. the control group (**a**, **b** scale bars = 100 μm)

After stimulation for 12 h, the number of cells passing through the Transwell pore in keratin group was approximately twice that of the control group (*P* < 0.01) (Fig. 4a). A similar result was observed during the wound-healing experiment, showing that damaged areas in the keratin group significantly decreased after 12 h (*P* < 0.001) (Fig. 4b).

**Fig. 5:**
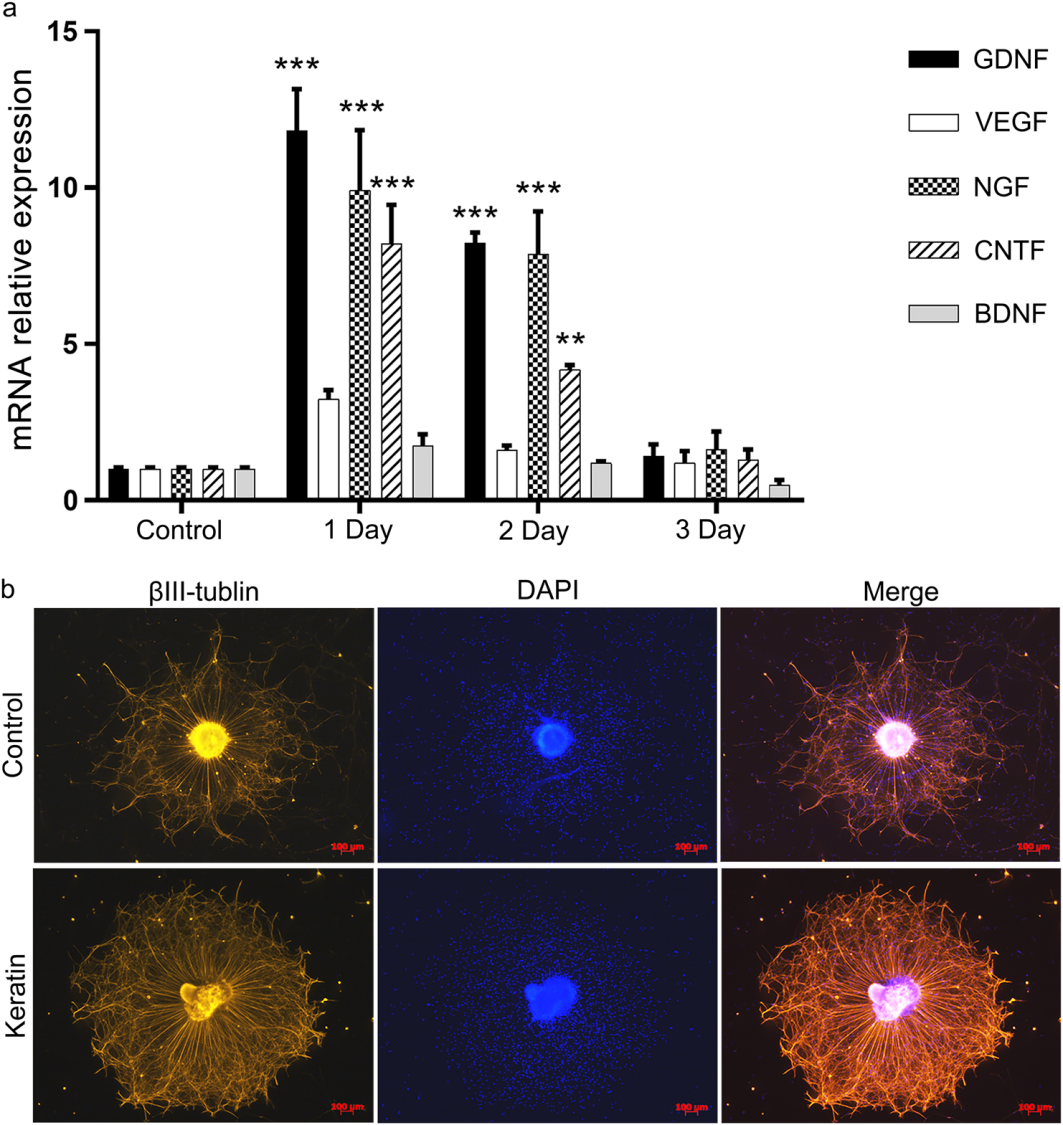
Keratin increased the neurotrophic factor expression of SCs and directly impacted the DRG axon growth. **a** Gene expression analysis of important neurotrophic factors (GDNF, VEGF, NGF, CNTF and BDNF) in RCS96 cells by RT-qPCR at 0, 1, 2 and 3 days. **b** Axonal regeneration of DRG after having been explanted on the control and keratin groups for 24 h. DRG was stained for βIII-tubulin and nuclei with DAPI. **P < 0.01, ***P < 0.001 vs. the control group (**b** scale bar = 100 μm)

Neurotrophic factors secreted by SCs play an important role in axon regeneration. The mRNA levels of GDNF, NGF and CNTF were much higher in keratin group on the first day. The secretion of neurotrophic factors decreases gradually with the prolongation of time, but the levels of GDNF, NGF and CNTF still exceeded those of the control group (Fig. 5a).

Immunofluorescent staining of the axon-specific marker βIII-tubulin was performed to detect the growth of DRG neurons. The axons in the keratin group were far superior to those in control group in terms of density and length of the neurofilaments after having been explanted for 24 h (Fig. 5b), indicating that keratin could indirectly participate in the repair of injury through activation of SCs but directly promote the extension of the axon.

### Keratin-adjusted LPS-induced inflammatory cytokine expression in RAW264.7 cells

**Fig. 6:**
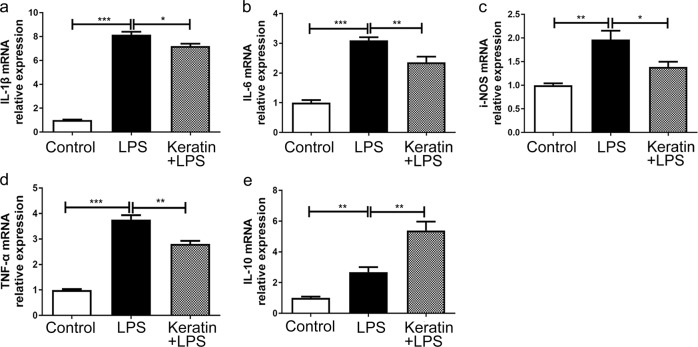
Effects of keratin on inflammatory cytokine-related gene expression in LPS-stimulated RAW264.7 cells. RAW264.7 macrophages were seeded on LPS and keratin groups for 24 h prior to LPS (1 μg/mL) treatments for 12 h. The mRNA levels of IL-1β (**a**), IL-6 (**b**), iNOS (**c**), TNF-α (**d**) and IL-10 (**e**) were quantitated by RT-qPCR. *P < 0.05, **P < 0.01, ***P < 0.001

After 12 h of LPS stimulation, the mRNA levels of inflammation-related factors IL-1β, IL-6, iNOS, TNF-α and IL-10 increased 2 to 4 times compared with the control group. However, the expressions of four pro-inflammatory genes in keratin group were reduced to some extent (Fig. 6a-d). Meanwhile, the mRNA level of anti-inflammatory factor IL-10 in keratin group was much higher than that in the LPS group (*P* < 0.01) (Fig. 6e).

### Sciatic nerve repair in vivo

**Fig. 7:**
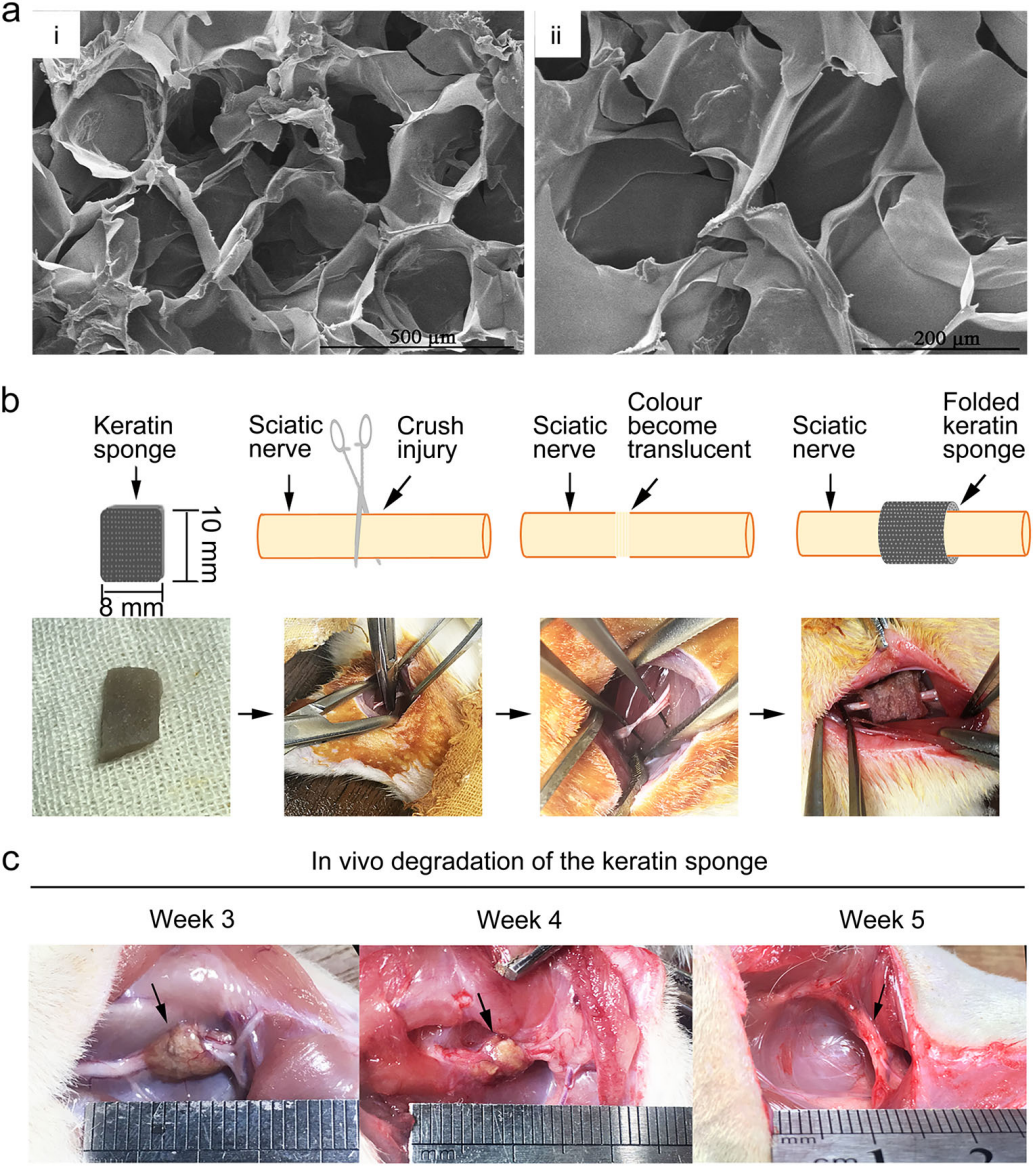
Repair of sciatic nerve injury by a keratin sponge in vivo. **a** SEM observation of keratin sponge and uniform microporous structure was conducted and the pore size was ≈200 μm in diameter. **b** The upper panel is a schematic diagram of surgery in the keratin group, and the lower panel shows the corresponding surgical photographs. **c** The degradation of keratin sponge was observed at weeks 3, 4 and 5 (**a** scale bars = 500 μm, **b** scale bars = 200 μm)

To further explore the repair effect of keratin on the peripheral nerve injury in vivo, we processed the keratin into a net-like keratin sponge with 200 µm pore size (Fig. 7a). As shown in the flow chart, the keratin sponge was wrapped around the injury site after the sciatic nerve crush (Fig. 7b). It can be observed in the fourth week after surgery that the keratin sponge was approximately half-absorbed by the body, and completely degraded within 5 weeks (Fig. 7c).

**Fig. 8:**
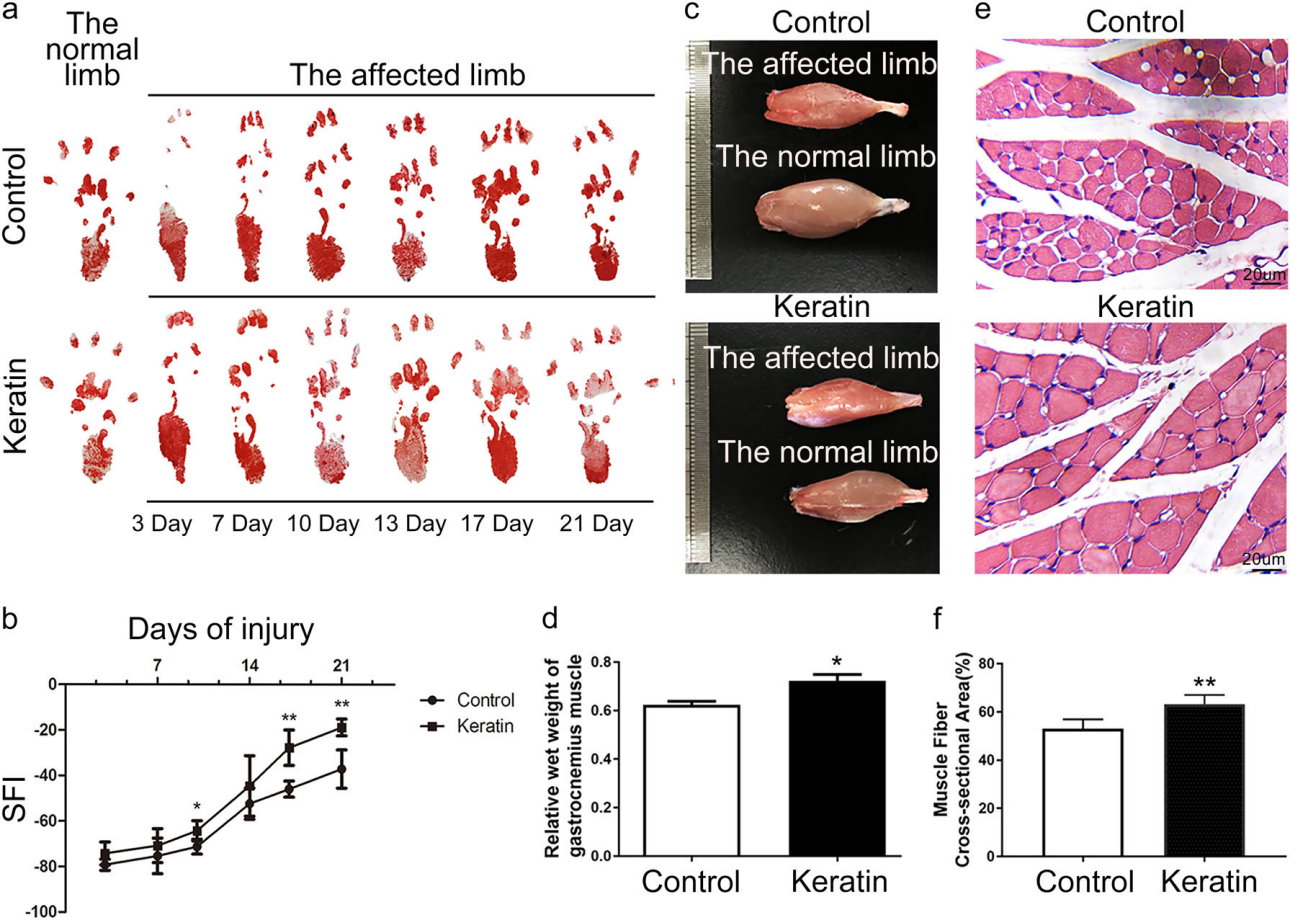
Footprints and gastrocnemius muscle analysis at 3 weeks after the injury of SD rats. **a** Footprint change at 3, 7, 10, 13, 17, and 21 days after surgery. **b** Sciatic nerve function index. **c** Macroscopic view of the gastrocnemius muscles. **d** Quantification and statistical analysis of wet weight ratios of gastrocnemius muscles. **e** HE staining of gastrocnemius muscles. **f** Quantification and statistical analysis of muscle fiber cross-sectional area. *P < 0.05, **P < 0.01 vs. the control group (**e** scale bars = 20 μm)

The footprints of the keratin group stretched at 10 days and approached the footprints of the normal limb at 21 days. The footprints of the control group began to change by day 13 and their recovery was less than that of the keratin group after 21 days (Fig. 8a).

Immediately following the sciatic nerve crush injury, SFI values dropped to –79.2 in control group and –74.2 in keratin group after 3 days, indicating a loss of sciatic nerve function. After 17 days, the keratin group showed greater functional improvement (−27.7) than the control group (−45.9) (*P* < 0.01), and the difference (−18.8 vs. −37.1) was more obvious at 21 days (*P* < 0.01) (Fig. 8b).

Compared with the keratin group, long-term denervation led to gastrocnemius atrophy and the wet weight of gastrocnemius muscle (*P* < 0.05) and diameter of muscle fibers (*P* < 0.01) decreased significantly in the control group at the 3rd week after injury (Fig. 8c–f).

**Fig. 9:**
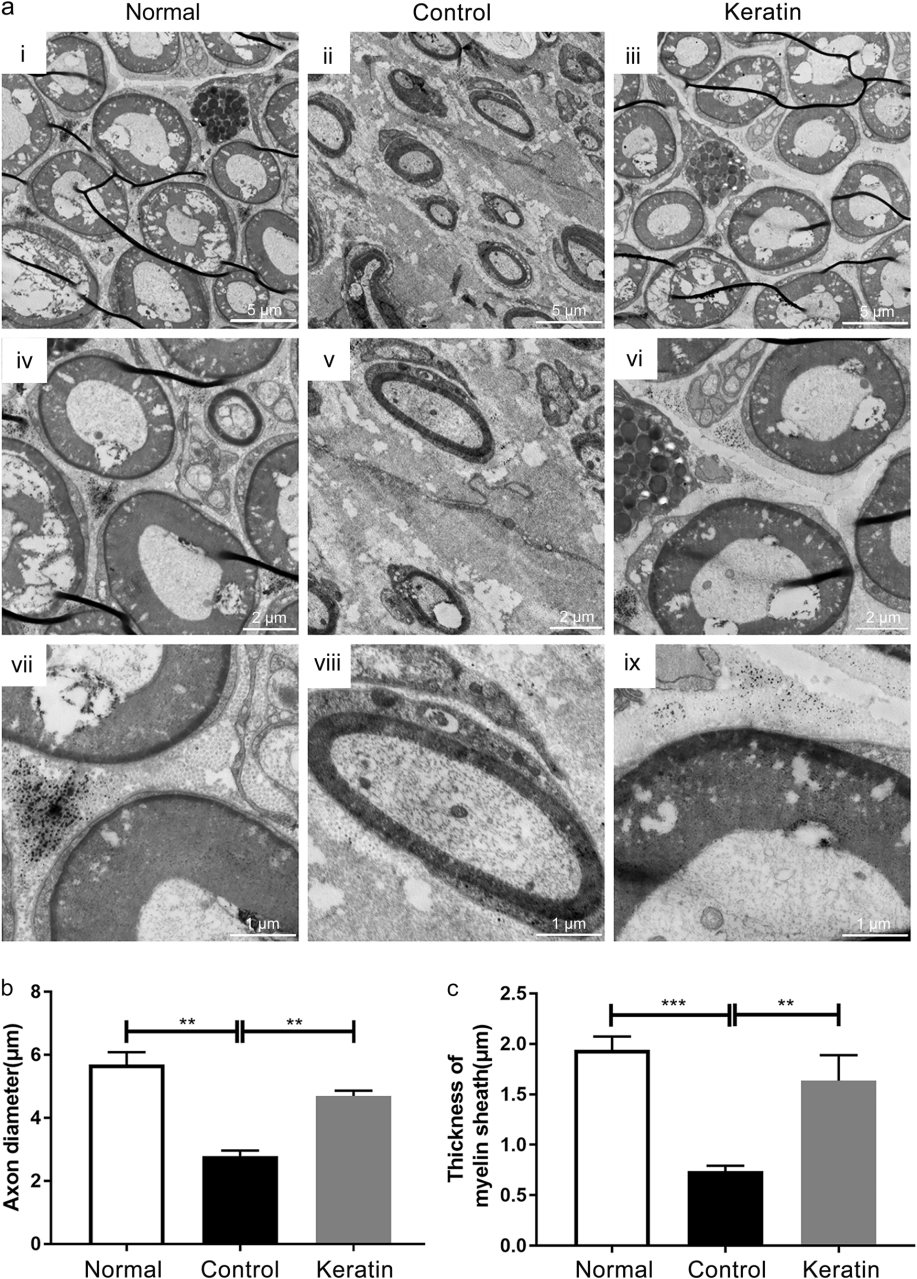
TEM observation of regenerated sciatic nerve fibers at 3 weeks after the injury of SD rats. **a** TEM observations of nerve cross-sections in three groups. **b** Quantification and statistical analysis of axon diameter. **c** Quantification and statistical analysis of myelin thickness. **P < 0.01, ***P < 0.001 (scale bar: **a**–**c** 5 μm, **d**–**f** 2 μm, **g**–**i** 1 μm)

The myelin morphologies of distal regenerative nerve in normal nerve group, control group and keratin group were observed by TEM (Fig. 9a). It was estimated that the keratin group was superior to the control group but close to the normal nerve group (*P* < 0.01) in both axon diameter and thickness (Fig. 9b, c).

## Discussion

The repair of peripheral nerve damage caused by natural disasters, car accidents or diseases remain a clinical difficulty. Without timely treatment, the target organ not connected with the regeneration nerve will gradually shrink, lose function and even result in disability [19, 20]. With the advancement of tissue engineering, some biological materials that promote nerve regeneration have been invented. Human hair keratin has been gradually applied to the field of nerve injury due to its good biological properties and plasticity. A high molecular weight keratin was obtained by opening disulfide linkages and destroying hydrogen bonds by reducing method [12]. This kind of keratin molecule has less negative charge in vivo and is apt to form a more stable structure [21].

As “repair cells” after injury, SCs play important roles in the re-establishment of neural structure. In vitro experiments showed that keratin had neuroinducible activity, and SCs grown in the keratin group had a series of changes in migration capacity, proliferation activity and morphology. These changes were similar to the activation of Epithelial-Mesenchymal Transition (EMT) program in SCs, which is the key physiological response of cells involved in repair [22]. SCs can also secrete neurotrophic factors to nourish axons, prevent axon apoptosis and stimulate cell proliferation by several pathways [23–25]. Our studies demonstrated for the first time that keratin can promote neuronal axon extension in vitro. The DRG neurons seeded on keratin group were superior to those of the control group in both quantity and length as demonstrated in Fig. 5b. This change may be related to the activation of intracellular cAMP levels by keratin, thereby increasing the intrinsic growth capacity of neurons [26].

SCs are not the only important cells in the regenerative environment. After nerve damage, macrophages are attracted to the site of injury, strictly regulate the local inflammatory response to avoid excessive tissue damage and create a suitable microenvironment for nerve regeneration [7, 21, 27]. LPS is a commonly used substance in the establishment of inflammatory models. The increase of anti-inflammatory factor IL-10 after LPS stimulation may reflect the protective immunity of cells. Keratin can downregulate the expression of pro-inflammatory factors and upregulate anti-inflammatory factors, as shown in Fig. 6. In addition, we observed that a small number of SD rats in control group showed inflammation reaction on the early postoperative, characterized by the redness and fever of toes. This body’s excessive inflammatory response to injury can recover spontaneously within a week. Due to the anti-inflammatory effect of keratin sponge, this phenomenon did not occur in the keratin group.

Ice crystals in keratin solution connected with each other at low temperature to form a homogeneous porous structure, which transformed keratin from liquid to solid. This spongy keratin has a porous structure and a certain mechanical strength, high temperature resistance and insolubility in water. It is well known that the pore structure plays an important role in the growth of new tissue and is beneficial to the nutrient and waste exchange and the inhibition of scar formation by fibroblasts [13, 28, 29]. The keratin sponge was wrapped around the lesion to provide a suitable space for nerve repair, preventing the adhesion to surrounding tissues and facilitating nerve regeneration. SD rats in the keratin group had significant improvement in terms of footprint analysis and SFI index compared with those in the control group from 14 days after surgery, suggesting that wrapping the keratin sponge in the injured site accelerates axon regeneration at an early stage, and lays a solid foundation for later functional recovery. The recovery of myelin sheath at the distal of the regenerated axons was further observed by TEM, the results of which showed that the axons in the keratin group are arranged neatly and that the diameter and thickness of the myelin sheath increase significantly, which is better than the control group and close to the normal nerve group. Because the axon cannot regenerate to the target organ in time or the regeneration effect is poor, the gastrocnemius wet weight and muscle fiber morphology in the control group were lower than those in the keratin group at 21 days. Studies have shown that axons grow at a rate of 1–3 mm/d after peripheral nerve injury, so the rate of degradation of keratin sponge should be adapted to the rate of axonal regeneration appropriately. In this study, most of the kinetic function of SD rats could be recovered in about 3–4 weeks after crush injury, so the degradation cycle of keratin sponge prepared in our laboratory is sufficient for nerve regeneration within 5 weeks.

Keratin has a wide range of applications and has been developed in other areas such as the repair of heart, skin and bone tissue. Previous studies have noted that an antibiotic-loaded keratin film shows high antibacterial effects in bacterial sensitivity assays and can potentially prevent wound infection [30]. In addition, keratin dressing applied to the wound site of animal models can accelerate the onset of epithelialization and faster wound healing compared with polyurethane dressing [31, 32]. However, the keratin still has some limitations in its application to sciatic nerve transection injury, so it must be combined with other materials to improve its mechanical strength. Nevertheless, we have reason to believe that keratin has great potential and prospect in the repair of peripheral nerve injury.

## Conclusions

In summary, the repair and regeneration after peripheral nerve injury is a complex process. Keratin, as a natural biomaterial, can regulate the biological activity of many kinds of cells, stabilize the microenvironment of the damaged site and accelerate the regeneration of axons. Therefore, keratin has a good promoting effect on both cell culture and tissue repair, but its potential regulatory mechanism needs to be further explored.
